# Inconclusive evidence for associations between adverse experiences in adulthood and working memory performance

**DOI:** 10.1098/rsos.241837

**Published:** 2025-01-08

**Authors:** Stefan Vermeent, Anna-Lena Schubert, Meriah L. DeJoseph, Jaap J. A. Denissen, Jean-Louis van Gelder, Willem E. Frankenhuis

**Affiliations:** ^1^ Department of Psychology, Utrecht University, Utrecht, The Netherlands; ^2^ Max Planck Institute for the Study of Crime, Security, and Law, Freiburg, Germany; ^3^ Department of Psychology, University of Mainz, Mainz, Germany; ^4^ Graduate School of Education, Stanford University, Stanford, CA, USA; ^5^ Department of Education and Child Studies, Leiden University, Leiden, The Netherlands; ^6^ Evolutionary and Population Biology, Institute for Biodiversity and Ecosystem Dynamics, University of Amsterdam, Amsterdam, The Netherlands

**Keywords:** working memory, adversity, deficits, developmental adaptation, structural equation modelling

## Abstract

Decades of research have shown that adversity tends to be associated with lower working memory (WM) performance. This literature has mainly focused on impairments in the capacity to hold information available in WM for further processing. However, some recent adaptation-based studies suggest that certain types of adversity can leave intact, or even enhance, the ability to rapidly update information in WM. One key challenge is that WM capacity and updating tasks tend to covary, as both types of tasks require the creation and maintenance of bindings in WM; links between mental representations of information in WM. To estimate the associations between adversity and different processes in WM, we need to isolate variance in performance related to WM capacity from variance in performance related to updating ability. In this Registered Report, participants from the Dutch Longitudinal Internet studies for the Social Sciences (LISS) panel completed three WM tasks: two complex span tasks and a task measuring both binding and updating of information. In addition, we estimated participants’ exposure to neighbourhood threat, material deprivation and unpredictability. We estimated associations between the three types of adversity and latent estimates of WM capacity and updating using structural equation modelling. We did not find consistent associations between adversity and WM capacity or updating, nor did we find evidence that the associations were practically equivalent to zero. Our results show that adversity researchers should account for overlap in WM tasks when estimating specific WM abilities.

## 1. Introduction

Living in adverse conditions, with prolonged exposure to intense stress, tends to have a profound and enduring impact on cognitive functioning [1–3]. Although adversity can be described in many ways, we follow contemporary models focusing on threat, deprivation and unpredictability as key dimensions of adversity [4–7]. A domain that seems to be particularly affected by adversity is working memory (WM). WM is a system for mentally building, maintaining and updating immediately relevant information [8]. Performance on WM tasks is associated with a host of social and cognitive abilities, such as math [9], reading [10], learning [11], general intelligence [12] and mentalizing [13]. Not surprisingly, then, deficits in WM have negative consequences for both educational and professional outcomes [14–17]. Decades of research show that adversity is generally negatively associated with performance on WM tasks [18]. However, emerging evidence suggests that specific aspects of WM might remain intact or even be enhanced through developmental adaptations to adversity. So far, the literature has tended to focus on related, but different aspects of WM in isolation, limiting a fuller integration. Here, we take a psychometric modeling approach to simultaneously examine potential decreases and enhancements in two WM components: capacity and updating.

### 1.1. Deficit-based and adaptation-based models

A large literature has shown negative associations between exposures to adversity and performance on WM tasks [1–3]. These associations may be potentially attributable to the enduring influence of stress on several key brain regions that support WM [19,20]. Much of this work has focused on WM capacity, or the ability to keep multiple pieces of information simultaneously available for further processing. For early-life adversity, this negative association is already present during childhood, and persists into adulthood ([1,18,21–24]; but see [25]). Studies with college students have found a link between both recent and lifetime experiences of stressful major life events (discrete negative events that have a clear onset and offset, unlike chronic adversity) with lower WM capacity [26–28].

The most common tasks used to examine the negative association between adversity and WM are simple span tasks (repeating a string of stimuli of increasing length), complex span tasks (remembering a string of stimuli while being engaged by a secondary task) and *n*-back tasks (judging whether the current stimulus in a string is identical to the stimulus *n* steps ago) [18]. Performance on these tasks is assessed through the number of items that participants can retain in WM, that is, their overall capacity (with the exception of *n*-back; for concerns about the construct validity of this task, see [29,30]).

Although both early-life and recent adversity appear to be negatively associated with WM capacity, a small set of studies suggest that exposure to adversity may leave intact, or even enhance, the ability to update items in WM in adolescents [31] and adults [32]. Updating is defined as the ability to rapidly replace old information in WM with new information. The finding that updating may be left intact or even enhanced after exposure to adversity exemplifies emerging theoretical frameworks grounded in adaptive reasoning that are complementary to deficit frameworks [5,33–35].

Adaptation-based theories assume that developmental processes tailor an individual’s cognitive abilities to the unique challenges and opportunities posed by their environment. The link between adversity and cognitive abilities is further assumed to be specific; as different types of adversity (e.g. threat versus deprivation) pose different challenges, they should (at least in part) shape cognitive abilities in different ways. For example, with regards to executive functioning, some previous studies have found that children and adults with more exposure to unpredictability (characterized by random variation in adversity exposure over space or time) and threat tend to be better at rapidly shifting their attention between tasks ([31,36–38]; but see [25]). WM updating may be especially adaptive in unpredictable environments. WM updating allows people to maintain an up-to-date overview of the (changing) current state of the environment [32]. Additionally, improved WM updating performance has also been documented for threat exposure [31]. An enhanced WM updating ability could facilitate keeping track of and integrating signals that may potentially signal acutely threatening situations.

### 1.2. Associations between WM capacity and updating

With deficit theories focusing on WM capacity and adaptation-based theories on WM updating, we may wonder how capacity and updating are related to each other. Performance on tasks measuring WM capacity and updating tends to be substantially correlated (in the range of 0.20 to 0.50 [39,40]). This overlap appears to stem from shared demands of both types of tasks, in particular the need to create and maintain arbitrary bindings [41–43]. The term *binding* refers to the process of mapping memory items to specific positions in WM (e.g. serial, spatial or temporal positions, depending on the task) [42,44]. For example, on most WM tasks, correct recall of memory items depends on remembering them in their correct serial position, or in relation to the location where they were presented.

The centrality of binding in WM is supported by theoretical models of WM and by empirical work showing that (latent) WM capacity is strongly related to the ability to maintain bindings [42–46]. The number of bindings a person can create and maintain in WM might be the main limiting factor in WM capacity, as maintaining several bindings at the same time will increasingly lead to interference between them [41–43]. This upper limit on WM capacity also affects performance on WM updating tasks. That is, updating items in WM requires not just dissolving old bindings and creating new ones, but also maintaining bindings of items that should not be updated. Thus, the overlap in performance on WM updating and capacity tasks likely stems from the need in both types of tasks to create and maintain bindings in WM [39,43,45,47,48].

Nevertheless, WM updating tasks additionally require the updating of established bindings, which sets them apart from WM capacity tasks [39,47]. Different updating tasks require different combinations of retrieval (making information available for immediate processing), transformation (changing a prior value into a new one, e.g. by addition or subtraction) and substitution (replacing a prior value for a new value) [47]. Ecker *et al*. [47] included three measures of WM capacity as well as eight versions of a WM updating measure that required different combinations of retrieval, transformation and substitution. After accounting for overall updating accuracy (which was positively correlated with WM capacity), they found positive correlations of around 0.50 between WM capacity with latent estimates of retrieval and transformation accuracy, but not with a latent estimate of substitution accuracy. Thus, when the ability to accurately substitute old with new information—a key aspect of WM updating—is sufficiently isolated from WM capacity using latent modelling, capacity and updating seem to be independent components of WM.

These findings underscore the importance of accounting for WM capacity when assessing a person’s WM updating ability. This is especially important in the context of adversity research, as previous studies suggest that certain types of adverse conditions might have opposing effects on WM capacity and updating (e.g. [18,31,32]). Yet, to our knowledge, no previous research has analysed both abilities within a single statistical model. This could lead to (i) an underestimation of the extent to which adversity undermines WM capacity and (ii) underestimation of the extent to which adversity can enhance WM updating. This, in turn, has implications for basic and applied science. For basic science, it could bias inferences about individual differences in performance on WM tasks, especially when the negative association between adversity and WM capacity is stronger than the positive association with WM updating. For applied science, it could hide from view potential pathways to leverage people’s existing strengths in school or work contexts.

### 1.3. Current study

In this study, we estimated associations between latent estimates of WM capacity and updating with three types of adversity: threat, deprivation and unpredictability. Together, these adversity types capture key dimensions in contemporary models of adversity [4–7]. Threat refers to experiences involving the potential for harm imposed by others. We focused on perceived neighbourhood violence, the extent to which an individual reports having been exposed to acts of violence in their neighbourhood. Deprivation refers to having a low level of resources. We focused on perceived material deprivation, a (perceived) lack of access to material resources. Unpredictability refers to variation in material deprivation over time. This definition is inspired by, but deviates from the harshness-unpredictability framework, in which unpredictability is defined as stochastic variation in harshness (age-specific rates in morbidity and mortality) over space and time [4,5]. We did not calculate unpredictability in neighbourhood threat given that participants had at most six timepoints, and often as few as one or two, which is insufficient to calculate variation over time [49].

We addressed three research questions. First, what is the association between adversity and WM capacity? Second, what is the association between adversity and WM updating *after* accounting for WM capacity? Third, are the directions and strengths of these associations similar or different for neighbourhood threat, material deprivation and unpredictability?

**Figure 1.**
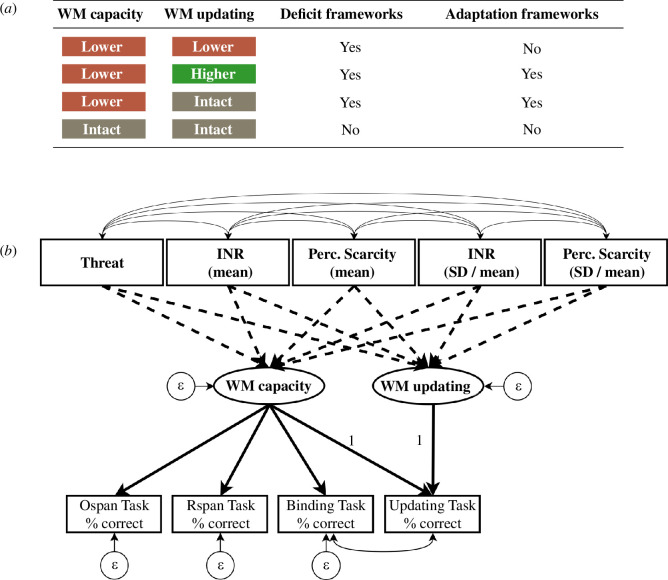
Overview of predictions derived from deficit and adaptation frameworks. Panel (*a*) depicts the most likely between-person data patterns based on previous literature, and whether we would consider them consistent with deficit and adaptation frameworks (see the main text for more details). Panel (*b*) depicts an overview of the preregistered Structural Equation Model. Note that this model differs slightly from the final model (see figure 4). Ellipses represent latent variables, rectangles represent manifest variables and circles represent residual variances. Unidirectional solid lines represent factor loadings, bidirectional solid lines represent covariances and dashed lines represent regression paths. All four manifest WM measures loaded on a latent WM capacity factor, reflecting the fact that people have to hold information active in WM on all tasks. We fixed the loading of WM capacity on the Binding Task to 1, reflecting the idea that the ability to create and maintain bindings is the main limiting factor in WM capacity [41–43]. WM updating was modelled as a latent factor capturing the residual variance in the updating task after accounting for variance related to WM capacity. INR = income-to-needs ratio; Perc. Scarcity = perceived scarcity; s.d. = standard deviation.

We evaluated evidence for deficit- and adaptation-based frameworks (see figure 1*a* for a visual summary, and electronic supplementary material, appendix 1 for the study design plan). Crucially, as deficit and adaptation processes can operate in concert [34], we could find support (or lack thereof) for both frameworks in the same model. We distinguished between three between-person data patterns: (i) lower performance, (ii) higher performance, and (iii) practically equivalent performance. We defined lower performance as a statistically significant negative association between a type of adversity and WM capacity or updating (irrespective of effect size). We defined higher performance as a statistically significant positive association between a type of adversity and WM capacity or updating (irrespective of effect size). We defined practically equivalent performance as an association between a type of adversity and WM capacity or updating that has a standardized effect smaller than 0.1 and larger than −0.1—even if the effect is statistically different from zero—which we tested using Two One-Sided T-Tests (TOST) equivalence testing (see §5.4.6; [50]).

Deficit frameworks predict a negative association between all three types of adversity and WM capacity as well as WM updating. This follows from the hypothesis that adversity leads to broad WM deficits [1,51]. Deficit frameworks are partially supported if we find negative associations with only one (or two) types of adversity.

Within adaptation-based frameworks, theories make two predictions. First, if adaptive processes enhance WM updating and there are no impairment processes operating, we can expect a positive association between adversity and WM updating. Second, if adaptive processes operate in concert with general impairment processes, we can expect practically equivalent WM updating performance in combination with lower WM capacity. If neither impairment nor adaptative processes are operating, we can expect both WM updating and capacity to be practically equivalent.

We also had two expectations based on prior studies. First, we expected the association between material deprivation and WM capacity to be more negative than the associations with unpredictability and neighbourhood threat. This follows from findings showing that cognitive abilities are more negatively associated with cognitive deprivation than threat [51,52]. Although cognitive and material deprivation are distinct types of deprivation, they tend to be correlated, and are both associated with limited access to resources that stimulate cognitive development and functioning [53–55]. Therefore, we expected that their associations with WM would have comparable effect sizes. Second, researchers have hypothesized that WM updating is particularly adaptive in unpredictable and threatening environments, as it facilitates keeping track of unpredictable changes and sudden threats. Therefore, we expected WM updating to be associated with unpredictability and neighbourhood threat, but not with material deprivation ([32]; but see [31]).

## 2. Methods

### 2.1. Participants

**Figure 2.**
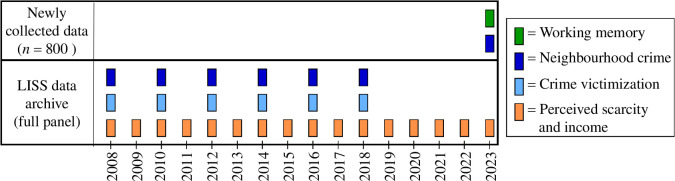
Overview of the different data sources used in this study. We distinguished between measures taken from the LISS data archive and measures that were newly collected in our own study between October 2023 and February 2024. Perceived scarcity and income were collected yearly in the full panel from 2008 to 2023. Neighbourhood crime and crime victimization were collected across six waves between 2008 and 2018. In the newly collected data, we collected data on a measure of neighbourhood threat and multiple measures of working memory. Note that participants did not have data across all timepoints of the archived studies because they joined the LISS panel more recently or because they did not participate in each wave.

Our study included 800 participant who were randomly sampled from the Longitudinal Internet studies for the Social Sciences (LISS) panel [56]. The LISS panel is a representative probability sample of roughly 5000 Dutch households (approx. 7500 individuals) drawn from the population register by Statistics Netherlands on an invite-only basis. Households without a computer or internet connection are provided with these facilities by LISS. Each year, participants complete the same core battery of questionnaires about—among other topics—their financial situation in the past year. In addition, participants can complete additional online questionnaires every month, with variable content. The current study integrated two data sources. First, our sample of 800 participants participated in a new LISS study between October 2023 and February 2024 (hereafter referred to as ‘newly collected data’), in which we included a measure of neighbourhood threat and multiple measures of working memory. Second, we accessed data that were previously collected in LISS (hereafter referred to as ‘the LISS archive’). See figure 2 for a visual overview of the data sources and their measurement timepoints. We signed a contract with LISS stipulating that we would receive access to the newly collected data only after Stage 1 acceptance of this Registered Report.

We based our power analysis on simulations reported by Kretzschmar & Gignac [57], determining the required sample size to detect a small effect size (β = 0.1) with at least 90% power at α = 0.05. Assuming a reliability of at least 0.7 (which is typical for WM tasks with a number of trials similar to ours; e.g. [43]), we required a sample size of *n* = 730. Anticipating some exclusions, we decided to include 800 participants. Participants were eligible for inclusion if they (i) were currently between 18 and 55 years old, (ii) had completed at least one wave of an archived longitudinal LISS study containing measures that we use to operationalize crime neighbourhood threat (see below), and (iii) had given permission to link their LISS data to government microdata (not relevant here).

To ensure sufficient representation of people from lower socioeconomic backgrounds, *half* the total sample was sampled from participants who reported one or more of the following at least once in the 3 years: (i) a monthly income < €1500, (ii) HAVO or VWO as highest completed education (which are the two highest levels in Dutch secondary education), or (iii) a score of 4 or lower on the ‘ladder of life’ (‘If you imagine a ‘ladder of life’, where the first step represents the worst possible life, and the tenth (top) step the best possible life, on what step would you place yourself?’). Participants were excluded if they (i) switched to and interacted with other browser tabs *during* one or more of the cognitive tasks, (ii) did not perform above chance level on the secondary processing tasks. The final sample consisted of 759 participants (see table 1).

**Table 1.** Descriptive statistics.

category	statistic
mean age (s.d.)	41 (9.9)
sex (% female)	54.4
highest completed education (%)	
primary school	0.5
vmbo (intermediate secondary education)	8.3
havo/vwo (higher secondary education)	9.2
mbo (intermediate vocational education)	26.4
hbo (higher vocational education)	31.5
wo (university)	22.4
other	0.5
missing	1.2
mean number of waves (s.d.)	
INR	13.4 (3.9)
perceived scarcity	11.1 (3.7)
threat	3.5 (1.9)

### 2.2. Measures

All independent variables, except for the income-to-needs ratio (INR), consisted of multiple items and/or scales. If all correlations between the items/scales were equal to or larger than 0.60 (i.e. indicating a ‘strong’ correlation), then we computed a uniformly weighted average. If the correlation was lower than 0.60, we applied Principal Component Analysis (PCA) to the averaged measures and extracted only the first principal component score. We present bivariate correlations in table 2, and histograms for all independent measures in the electronic supplementary materials.

**Table 2.** Spearman correlation between the main independent variables. CV = coefficient of variance, INR = income-to-needs ratio, M = mean, Perc. Scarcity = perceived scarcity.

	1	2	3	4	5	6	7	8	9	10	11	12	13	14
INR (M)	—													
living off income (M)	−0.52***	—												
financial troubles (M)	−0.43***	0.71***	—											
current situation (M)	−0.51***	0.75***	0.69***	—										
perceived scarcity (M)	−0.55***	0.96***	0.79***	0.89***	—									
INR (CV)	−0.17***	0.15***	0.21***	0.14***	0.18***	—								
living off income (CV)	0.19***	−0.20***	−0.02	−0.18***	−0.19***	0.15***	—							
financial troubles (CV)	−0.36***	0.64***	0.92***	0.60***	0.71***	0.24***	0.05	—						
current situation (CV)	0.20***	−0.11**	0.04	−0.11**	−0.11**	0.12**	0.34***	0.13***	—					
perceived scarcity (CV)	0.21***	−0.11**	0.05	−0.16***	−0.11**	0.18***	0.35***	0.16***	1.00***	—				
neighbourhood safety	−0.13***	0.19***	0.16***	0.17***	0.20***	0.05	−0.10*	0.12**	−0.05	−0.05	—			
neighbourhood violence Scale	−0.22***	0.26***	0.19***	0.22***	0.27***	0.02	−0.10*	0.16***	−0.06	−0.05	0.24***	—		
crime victimization	0.01	0.12**	0.18***	0.16***	0.15***	0.10**	0.01	0.17***	0.07	0.07	0.06	0.12**	—	
threat	−0.21***	0.29***	0.24***	0.26***	0.31***	0.07	−0.12**	0.20***	−0.06	−0.04	0.58***	0.89***	0.26***	—
mean	1.99	4.17	1.30	2.35	2.60	0.22	0.27	0.21	0.27	−0.01	1.45	2.39	1.04	−0.02
s.d.	0.76	1.60	0.53	0.75	0.87	0.19	0.17	0.24	0.15	0.99	1.47	0.95	1.27	0.68
min	0.09	1.00	1.00	1.00	1.00	0.01	0.00	0.00	0.00	−1.86	0.00	1.00	0.00	−1.07
max	6.10	11.00	4.44	5.00	5.93	1.52	0.99	0.92	0.93	4.42	8.00	6.86	7.00	3.68
skew	1.06	0.76	2.47	0.44	0.87	2.31	0.95	0.62	0.22	0.34	1.18	1.33	1.28	1.21
kurtosis	3.42	1.39	6.86	−0.08	1.08	8.83	1.22	−1.01	0.87	1.34	1.22	2.35	1.27	2.05

* = *p* < 0.05, ** = *p* < 0.01, *** = *p* < 0.001.

#### 2.2.1. Neighbourhood threat

#### Perceived neighbourhood crime

We included four items from the LISS archive collected across six waves (https://doi.org/10.17026/dans-zch-j8xt), in which participants answered how often it happens that they (i) ‘avoid certain areas in your place of residence because you perceive them as unsafe’, (ii) ‘do not respond to a call at the door because you feel that it is unsafe’, (iii) ‘leave valuable items at home to avoid theft or robbery in the street?’, (iv) ‘make a detour, by car or on foot, to avoid unsafe areas’ on a scale of 1 (‘(Almost) never’), 2 (‘Sometimes’) or 3 (‘Often’). We recoded these items so that 0 indicated ‘(Almost) never’. We then summed the responses within each wave for which participants had data, and calculated an average across the waves.

In addition, we implemented the Neighbourhood Violence Scale [34,58] in the newly collected data. The Neighbourhood Violence Scale includes seven items measuring perceived exposure to neighbourhood violence (e.g. ‘Crime is common in the neighbourhood where I live’; ‘Where I live, it is important to be able to defend yourself against physical harm’). Participants answered these questions on a scale of 1 (‘Completely disagree’) to 7 (‘Completely agree’). We computed an average of the seven items.

#### Crime victimization

We used data from the LISS archive collected across six waves (same dataset as above), in which participants indicated whether they fell victim to eight types of crime over the 2 years prior to a particular wave (0 = no, 1 = yes). We included seven items concerning exposure to crime: (i) burglary or attempted burglary; (ii) theft from their car; (iii) theft of their wallet or purse, handbag or other personal possession; (iv) wreckage of their car or other private property; (v) intimidation by any other means; (vi) maltreatment of such serious nature that it required medical attention; (vii) maltreatment that did not require medical attention. We computed a variety score by summing the exposures to *unique* types of crime across all waves. Thus, if a participants reported exposure to the same type of crime on separate waves, this counted as one exposure in the total score [59].

#### Neighbourhood threat composite

We first computed an average across time for each measure separately (i.e. the two measures of neighbourhood crime and the measure of crime victimization). Because correlations were below 0.60 (see table 2), we then used PCA to extract only the first principal component score (R^2^ = 0.20). The threat component was most strongly determined by the Neighbourhood Violence Scale (0.63), followed by the perceived neighbourhood crime scale from the LISS archive (0.40) and crime victimization (0.18).

#### 2.2.2. Material deprivation

We measured material deprivation with two separate indicators: perceived scarcity and the income-to-needs ratio.

#### Perceived scarcity (mean)

We used a few items from the LISS archive that were collected on a yearly basis between 2008 and 2023 (https://doi.org/10.57990/1gr4-bf42) to index perceived scarcity. First, participants indicated how hard or easy it currently is to live off the income of their household, on a scale from 0 (very hard) to 10 (very easy). Second, participants were asked to choose which of the following best applied to their current situation: (i) ‘we are accumulating debt’; (ii) ‘we are somewhat eating into savings’; (iii) ‘we are just managing to make ends meet’; (iv) ‘we have a little bit of money to spare’; (v) ‘we have a lot of money to spare’. Responses were reverse-coded, so that higher scores indicated a worse financial situation. Third, participants answered which of the following issues they were confronted with at present (0 = no, 1 = yes): (i) ‘having trouble making ends meet’; (ii) unable to quickly replace things that break’; (iii) ‘having to lend money for necessary expenditures’; (iv) ‘running behind in paying rent/mortgage or general utilities’; (v) ‘debt collector/bailiff at the door in the last month’; (vi) ‘received financial support from family or friends in the last month’.

We first computed the average across time for each item separately. Because correlations were all above 0.60, we calculated a uniformly weighted average.

#### Income-to-needs (mean)

We calculated an income-to-needs ratio for each year using monthly self-reported net household income from the LISS archive (https://doi.org/10.57990/qn3k-as78). Zero values in household income were set to missing, as these could either indicate the lack of an income or an unwillingness to disclose the income. If monthly household income is missing (or zero) for an entire year for a participant, we used, if available, the yearly net household income they reported in the LISS archive (https://doi.org/10.57990/1gr4-bf42), dividing it by 12 to obtain a monthly estimate. First, we divided the average income per year by the *poverty threshold*, as determined by Statistics Netherlands ([60]; CBS, personal communication, 15 December 2023). As thresholds are only provided for households with up to three children, we applied the threshold of a household with three children to households with more than three children. Likewise, we applied the threshold of a household with two adults for households that contained three or more adults. Second, we calculated the average within-person income-to-needs ratio for each year by averaging across the monthly income-to-needs estimates.

#### 2.2.3. Unpredictability

#### Perceived scarcity (s.d./mean)

This measure was based on the same items as outlined above (see ‘Perceived scarcity (mean)’). We computed unpredictability over time in perceived scarcity using the coefficient of variation, which is the within-person standard deviation across years divided by the mean [49,61–64]. The mean and standard deviation in income have been found to be strongly positively correlated, indicating that people with lower incomes tend to experience less variability in income [65,66]. For that reason, the standard deviation alone has been called into question as a measure of adversity, as the same fluctuation in income can have a greater relative impact for people close to the poverty line than for people with high incomes.

We first computed the coefficient of variation across time for each item separately. Because correlations were below 0.60 (see table 2), we then used PCA to extract only the first principal component score (R^2^ = 0.38). The perceived unpredictability component was almost fully determined by the item about people’s current situation (1.00), followed by difficulties to live off income (0.34) and financial troubles (0.20).

#### Income-to-needs (s.d./mean)

Similar to perceived scarcity, we computed unpredictability over time in the income-to-needs ratio using the coefficient of variation.

#### 2.2.4. WM tasks

The WM tasks were all part of the newly collected data. All materials and scripts for the cognitive tasks can be found at https://stefanvermeent.github.io/liss_wm_profiles_2023/materials/README.html. Prior to collecting LISS data, we conducted a pilot study in a Dutch sample (*n* = 100) through Prolific Academic. The main goals of this pilot study were to collect participant feedback (e.g. difficulty of instructions, whether we included sufficient breaks) and to analyse performance and correlations between tasks. The results of this pilot study are described in more detail in the electronic supplementary materials (https://stefanvermeent.github.io/liss_wm_profiles_2023/supplement/README.html).

#### Operation Span Task

**Figure 3.**
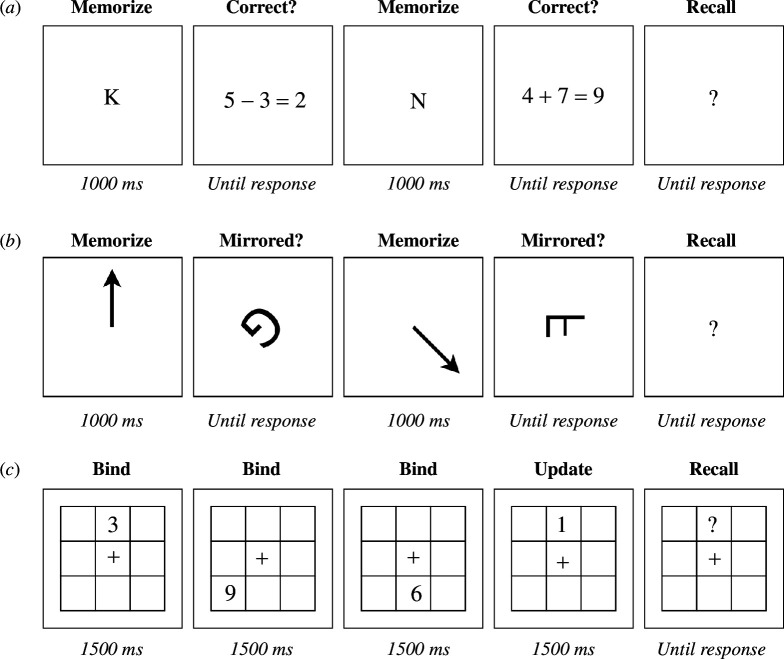
Overview of the working memory tasks. Panel (*a*): Operation Span Task. Participants memorized letters in the correct order, while engaging in a secondary math task. Panel (*b*): Rotation Span Task. Participants memorized the orientation of arrows, while judging whether letters were mirrored or normal in a secondary task. Panel (*c*): Participants memorized numbers in the correct location in a 3×3 grid. On half of the trials, all numbers were presented in unique locations, only requiring binding the numbers to the correct position. On the other half, some numbers were presented in the same location as a previously presented number, requiring updating. Note: stimuli are not to scale.

The Operation Span Task (figure 3*a*) is a common measure of WM capacity [43,67]. In this task, participants alternate between a primary memorization task and a secondary processing task. On each trial, the task is to memorize a sequence of letters in the correct order (from a set of 12 letters). Each letter is presented for 1000 ms in the centre of the screen. Next, participants see a simple mathematical equation including the outcome. Their task is to indicate whether the outcome is correct or incorrect by pressing either the ‘a’ or ‘l’ key on their keyboard. The equations always contain one addition or subtraction, with numbers ranging between 1 and 10. Outcomes are always positive integers. On each trial, participants have to memorize between four and six letters, with each set size repeated three times. At the end of each sequence, all letters are presented in a 3×4 grid, and participants click the letters in the correct order.

Participants first practiced the letter task (three times), then the math task (eight times) and then the full task (three times). If they performed at or below chance, they had the opportunity to either repeat a part or advance to the next part. After practising, participants completed 9 test trials, with a total of 45 recall items and 45 math items. We computed an operation span score by calculating the proportion of letters recalled in the correct sequential position across trials [67].

#### Rotation Span Task

The Rotation Span Task (figure 3*b*) is similar to the Operation Span Task and was adopted from Wilhelm *et al*. [43]. On each trial, the task is to memorize the orientation of a sequence of arrows in the correct order. Arrows can take on eight different orientations, with increments of 45°. Each arrow is presented for 1000 ms in the centre of the screen. Next, participants see a capital ‘G’ or ‘F’ that is rotated at one of eight different orientations, with increments of 45°. Their task is to indicate whether the letter is mirrored or not. On each trial, participants have to memorize between two to five arrows, with each set size repeated three times. At the end of each sequence, all arrows are presented simultaneously, and participants click the arrows in the correct order.

Participants first practised the arrow task (three times), then the letter task (eight times) and then the full task (three times). If they performed at or below chance, they had the opportunity to either repeat a part or to advance to the next part. After practising, participants completed 12 test trials, with a total of 45 recall items and 45 letter items. We computed a rotation span score by calculating the proportion of arrows recalled in the correct sequential position across trials [67].

#### Binding-Updating task

The Binding-Updating task (figure 3*c*) was adopted from Wilhelm *et al*. [43]. On each trial, participants see a 3 × 3 grid, with a fixation cross in the central cell. After 1000 ms, they are presented with a sequence of numbers (0–9) in random locations of the grid. Each new number is presented for 1500 ms, after which it disappears for 500 ms before the next number is presented. The task is to remember the last number they see in each location. Memory set sizes (i.e. the number of unique locations in the grid) ranges between three and five. On half of the trials, only one number is presented in each location. These constitute the binding trials. On the other half of the trials, some letters are presented in the same location as previous numbers, requiring mentally replacing the old number with the new number. These constitute the updating trials. We use two, three and four updating steps, each repeated in combination with the different set sizes. At the end of the trials, participants indicate which letter they saw last in each location in random order.

Participants first completed four practice trials. If they performed at or below chance, they had the opportunity to either repeat the practice trials or to advance to the actual task. After practising, they completed 18 test trials, of which nine were binding-only (24 recall items in total) and nine were updating trials (24 recall items in total). We computed a binding score by calculating the overall recall accuracy (%) across trials with zero updating steps. We computed an updating score by calculating the overall recall accuracy (%) across trials with one or more updating steps.

### 2.3. Procedure

We received ethical approval from the first author’s institutional ethical board. Upon starting the study, participants were informed that the study could only be completed on a laptop or desktop PC. If they attempted to start the study on a tablet or smartphone, they were unable to advance and prompted to switch to a suitable device. Participants started with the WM tasks, which on average took between 20 and 25 min. The WM tasks were completed in fullscreen mode. If participants left fullscreen mode at any moment during the tasks, they saw instructions at the top of their screen that allowed them to return to fullscreen mode. The order of the WM tasks was counterbalanced, and participants had the opportunity to take breaks at regular intervals.

After the cognitive tasks, participants answered three questions about the environment in which they completed the WM tasks: (i) ‘How much noise was there in your environment during the memory tasks?’; (ii) ‘Were you at any moment interrupted during the memory tasks?’; (iii) ‘Did you at any moment during the memory tasks leave the computer?’ Next, they completed questionnaires about their future orientation (not considered here), personality (not considered here), past adversity exposure and recent adversity exposure. Finally, they completed a standard set of evaluation questions asking about their experiences with the study, with the possibility to provide open-ended feedback. This part on average took 5 min. Participants received €7.50 for their participation through LISS. If participants experienced difficulties of any sort, they could contact the LISS helpdesk.

### 2.4. Proposed analysis plan

The Stage 1 protocol of this Registered Report can be found at https://osf.io/dp7wc.

#### 2.4.1. Data access

The working memory data and one of the neighbourhood threat indices were collected through October–December 2023, prior to submitting the Stage 1 protocol. These data were only made available to the first author after Stage 1 acceptance, as stipulated in a signed contract with LISS. During planning of the study, the first author accessed the LISS data archive and inspected three waves of the LISS data containing the items about neighbourhood safety and crime exposure, as well as the three most recent monthly data collections containing basic demographic info. The purpose was to ascertain the number of individuals who had finished the previous waves in the LISS data archive and were presently still participating in the panel (i.e. to see if we could reasonably create a link between the LISS data archive and newly collected data).

All data access events were automatically detected and logged on the GitHub repository using the *projectlog* R package [68]. We took the following measures to prevent bias: (i) we randomly shuffled the participant IDs in each dataset using the *projectlog* R package, so that we were unable to link participant data between (waves of) studies in the LISS data archive; (ii) we did not inspect any of the measures that would be part of our adversity composites; (iii) we did not know which participants would be selected for the newly collected data; (iv) the primary analyses were based on composite measures that combined measures from the LISS data archive with measures from the newly collected data.

#### 2.4.2. Primary analyses

See figure 1*b* for an overview of the model specification. We fitted a single model containing all adversity measures using the *lavaan* R package [69]. We used robust maximum likelihood estimation to account for non-normality. Missing data were handled using full information maximum likelihood (FIML). We accounted for clustering within families using the *lavaan.survey* R package [70].

WM capacity was estimated as a latent factor loading on all outcome measures. In addition, we estimated WM updating as a latent factor capturing residual variance in the updating measure. Thus, this factor accounted for updating-specific variance after accounting for WM capacity. We estimated the effect of each adversity type (dashed lines in figure 1*b*) through regression analyses. Each association was controlled for (i) age in years; (ii) the quadratic effect of age; (iii) environmental noise (‘How noisy was your environment during the memory tasks’, rated on a scale of 1 (very little noise) to 5 (a lot of noise)); (iv) two items measuring interruptions (‘Were you at any moment interrupted during the memory tasks?’ and ‘Did you at any moment during the memory tasks leave your computer?’, rated as yes or no). Goodness of fit was assessed using the comparative fit index (CFI) and the root mean square error of approximation (RMSEA). CFI values >0.90 and RMSEA values <0.08 were interpreted as acceptable model fit, and CFI values >0.95 and RMSEA values ≤0.06 as good model fit [71].

We anticipated that we may have to optimize the model further in case of bad model fit, and therefore planned to estimate the model in two steps to prevent bias. First, we constructed the measurement model of WM, without including the adversity measures. This step was planned to be carried out prior to accessing any of the adversity measures. Once we obtained at least acceptable model fit, we accessed and added the adversity measures to the model. This procedure was tracked and timestamped on the GitHub repository using the procedure outlined above. We controlled for multiple testing using the false discovery rate [72,73].

To statistically test whether small effects were practically equivalent to zero we used Two One-Sided T-tests (TOST) equivalence testing [50], using −0.1 and 0.1 as equivalence bounds. TOST equivalence testing allows us to conclude practically equivalent performance based on a significant effect, rather than erroneously interpreting a non-significant effect as evidence for the absence of an effect. We considered any effect that fell within this region to reflect practical equivalence, that is, a between-person difference in performance that is practically equivalent to zero. TOST provides two *p*-values, one testing against the upper bound and one testing against the lower bound; we report only the largest of the two *p*-values.

## 3. Results

### 3.1. Confirmatory analyses

#### 3.1.1. Model fit

**Figure 4.**
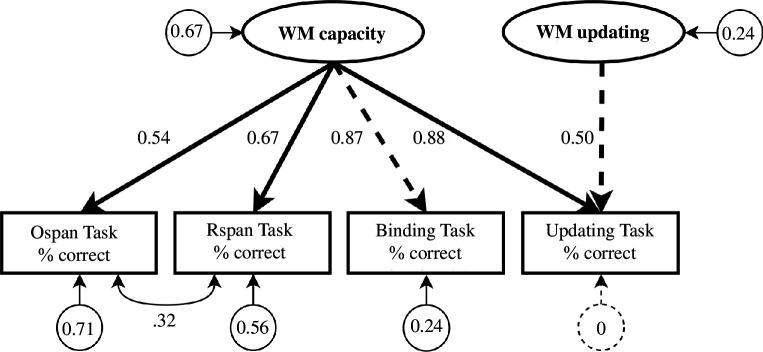
Overview of the final measurement model of WM performance. Ellipses represent latent variables, rectangles represent manifest variables and circles represent unstandardized residual variances. Unidirectional lines represent standardized factor loadings and bidirectional lines represent covariances. All four manifest WM measures loaded on a latent WM capacity factor, reflecting the fact that people have to hold information active in WM on all tasks. We fixed the loading of WM capacity on the Binding Task to 1, reflecting the idea that the ability to create and maintain bindings is the main limiting factor in WM capacity [41–43]. WM updating was modelled as a latent factor capturing the residual variance in the updating task after accounting for variance related to WM capacity. WM = working memory; Ospan = Operation Span; Rspan = Rotation Span.

The preregistered measurement model specification did not converge. A model version excluding the covariance between manifest binding and updating did converge, but resulted in suboptimal fit (Robust CFI = 0.95, Robust RMSEA = 0.12, 95% CI = [0.09, 0.14]). Modification indices indicated that model fit would improve most from estimating the covariance between Rotation Span and Operation Span, which is in line with previous factor models of working memory containing span tasks as a subset of other working memory tasks (e.g. [40]). A model incorporating an estimate of this covariance provided a good fit to the data (Robust CFI = 1, Robust RMSEA = 0, 95% CI = [0, 0]). After finalizing the measurement model, we constructed the final structural model by adding all predictors and covariates to the model, which resulted in a good model fit (Robust CFI = 0.99, Robust RMSEA = 0.03, 95% CI = [0, 0.03]). Figure 4 presents a visual overview of the final model.

#### 6.1.2. Associations between adversity and WM

**Figure 5.**
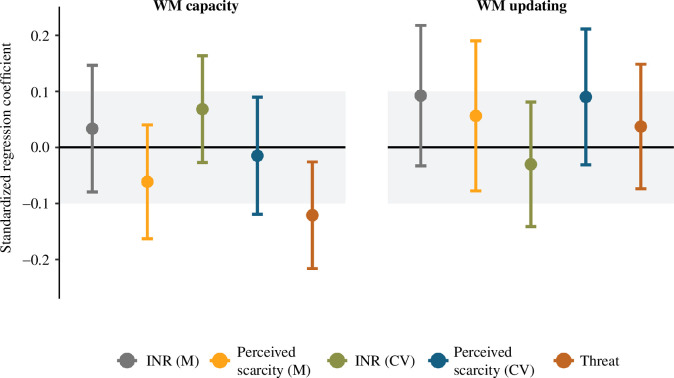
Results of the structural part of the SEM model testing the association between threat, deprivation and unpredictability on latent estimates of WM capacity and WM updating. The grey area shows the area of practical equivalence. Solid points indicate effects outside the area of practical equivalence, which was true for all effects. Standard errors represent the 95% confidence intervals. CV = coefficient of variation; INR = income-to-needs ratio; M = mean; WM = working memory.

The main results of the associations between the adversity measures and WM are summarized in figure 5. None of the adversity measures were significantly associated with WM capacity after adjusting for multiple testing (all *p*s ≥ 0.063). We also did not find evidence for practical equivalence for associations between any of the adversity measures and WM capacity (all *p*s ≥ 0.055). Similarly, none of the adversity measures were significantly associated with WM updating after adjusting for multiple testing (all *p*s ≥ 0.370). We also did not find evidence for practical equivalence to zero for associations between any of the adversity measures and WM updating (all *p*s ≥ 0.109).

### 6.2. Post hoc non-preregistered analyses

We conducted three post hoc non-preregistered analyses, described in more detail in the electronic supplementary materials. First, to contextualize our findings based on latent WM estimates, we estimated associations between adversity and performance on the separate WM tasks using four linear regressions. Threat had small, significant negative associations with performance on the Rotation Span Task (β = −0.13, *p* = 0.002), Operation Span Task (β = −0.14, *p* = 0.002) and Binding Task (β = −0.12, *p* = 0.004). None of the types of adversity were significantly associated with performance on the Updating Task (all *p*s > 0.181), and only the association with unpredictability in the income-to-needs was practically equivalent to zero (*p* = 0.041).

Second, the inconclusive nature of our confirmatory results could indicate that the true effect sizes were smaller than the effect size of interest that we used for our power analysis (β = 0.1; i.e. that we lacked sufficient power). To explore this, we conducted an alternative test for the absence of an association between adversity and WM by constraining regression paths between adversity and WM factors to zero in the SEM. Constraining all paths to latent WM capacity to zero significantly reduced model fit, although the change in AIC was below the cut-off as proposed by Burnham & Anderson [74], ΔAIC = 7.62, Δχ(5) = 14.20, *p* = 0.014, Robust CFI = 0.99, Robust RMSEA = 0.03, 95% CI = [0.01, 0.04]. Constraining all paths to latent WM updating did not significantly reduce model fit, ΔAIC = 3.81, Δχ(5) = 5.85, *p* = 0.321, Robust CFI = 0.99, Robust RMSEA = 0.03, 95% CI = [0, 0.03]. Thus, these results were somewhat inconsistent with the preregistered frequentist equivalent tests.

Third, as a non-preregistered robustness check, we calculated Bayes factors for the preregistered equivalence tests using the *bain* package [75], in which we evaluated whether the observed data are more likely under the hypothesis that the effects fall within the equivalence bounds, relative to the hypothesis that the effects fall outside of the equivalence bounds. The results are summarized in electronic supplementary material, table S3. For all but one association, the model comparisons showed at least strong evidence in favor of the data being more likely under the hypothesis that the effects fell within the equivalence bounds (BF_10_ ranging between 16.9 and 158.9). The only exception was the association between threat and WM capacity, for which we found moderate evidence in favour of the data being more likely under the hypothesis that the effect fell within the equivalence bounds (BF_10_ = 5.5). Thus, these results were inconsistent with the preregistered frequentist equivalent tests, which did not find evidence for practical equivalence.

### 3.3. Deviation from the Stage 1 protocol

In the Stage 1 protocol, we planned to first access the dependent variables to construct the SEM, and then access the independent variables. Due to an unintended error, the first author already accessed the datasets containing the measures that would be used to compute the independent variables before finalizing the SEM. However, beyond reading them into the R environment, these data were not yet inspected, manipulated or summarized. We contacted the PCI recommender upon finding out about this deviation, and agreed to describe this deviation as done here. For the sake of transparency, we timestamped the scripts for processing the independent variables at the moment of this unintended data access (https://github.com/StefanVermeent/liss_wm_profiles_2023/blob/d143e551018ba27313643a15bed57f329974272d/scripts/2_pipeline/1_ivs.R). They contain the code to read in the data, but no code yet for any type of data cleaning or variable computation.

## 4. Discussion

We investigated associations between adversity (threat, material deprivation and unpredictability) and WM capacity, a person’s ability to hold information available for later processing, as well as WM updating, a person’s ability to mentally replace old with new information. We distinguished between WM capacity and updating on a latent level using four different tasks, three of which are primarily construed as WM capacity tasks, and one that is primarily construed as a WM updating task. The WM capacity factor loaded on performance of all four tasks, in line with previous findings [39,41–43]. An additional WM updating factor accounted for the portion of variance in the Updating Task that was not explained by WM capacity. We did not find any consistent associations between adversity and WM capacity nor updating in our preregistered analyses. On the one hand, none of the associations significantly differed from zero. On the other hand, none of the associations fell within the pre-specified region of practical equivalence to zero (i.e. a between-person difference in performance that is practically equivalent to zero).

The confirmatory results were not consistent with hypotheses generated from a deficit framework. A large literature has documented negative associations between exposure to early-life adversity—especially deprivation—and WM capacity, which persists into adulthood ([1–3,18,32]; but see [25]). Similarly, studies with young adults have found that a higher frequency of recent as well as lifetime stressful major life events (i.e. negative events with a clear onset and offset, unlike chronic adversity) is also negatively associated with WM capacity [26–28]. The results were also not consistent with hypotheses generated from adaptation frameworks. Recently, a small set of studies documented intact and even higher WM updating performance in adolescents and adults who reported more exposure to childhood adversity [31,32]. These associations have been interpreted as reflecting developmental adaptations to adversity: in more threatening and unpredictable environments, it may be beneficial to be able to rapidly update the items held in WM [5,33–35]. By contrast, we did not find consistent associations between adversity exposure and WM updating. These findings are inconclusive, as we also did not find evidence for practical equivalence in our preregistered analysis.

A set of non-preregistered robustness checks were comparatively more consistent with practically equivalent performance, although they did not fully rule out the existence of small associations between adversity exposure and working memory performance. First, a Bayesian reanalysis of the preregistered equivalence tests (using the same equivalence bounds) provided strong evidence in favour of the hypothesis that working memory performance was practically equivalent, in contrast to the preregistered analyses. Second, constraining the regression paths in the SEM to zero somewhat reduced model fit for WM capacity, but not for WM updating. This suggests that there may have been systematic associations with WM capacity that were smaller than the equivalence bounds used in the (Bayesian) equivalence tests. If true, the associations would be smaller than we expected based on the literature outlined above, and would require a larger sample size to reliably detect. These analyses were not part of the registered analysis protocol, and therefore should be interpreted with sufficient caution pending replication.

The Updating Task shared a large proportion of variance with the WM capacity measures, which aligns with prior psychometric work focused on the structure of WM [43,45,76]. This highlights an important methodological issue for the field of adversity research, especially researchers working from adaptation frameworks, who hypothesize distinct effects of adversity on different components of WM (in contrast to deficit-oriented researchers, who predict adversity to have a negative effect on all components of WM). Specifically, adaptation-oriented researchers have hypothesized that certain types of adversity may enhance WM updating through developmental adaptation, while impairing WM capacity [5,31,32]. So far, this hypothesis has—to our knowledge—only been tested based on raw performance on single WM updating tasks. However, if true, performance on single WM updating tasks may substantially underestimate positive associations between adversity and WM updating, as raw performance may be influenced by both deficit and adaptation processes (the former influencing WM capacity, inadvertently measured in WM updating tasks). Leveraging these psychometric insights will be pivotal to better understanding associations between adversity and WM for future studies.

Aside from psychometric considerations, a second potential reason for the discrepancy between our findings and those from previous studies is that our investigation focused on adverse experiences in adulthood. By contrast, most previous studies have focused on the effects of either childhood adversity or stressful life events. It is possible that, relative to childhood adversity, the association between adversity in adulthood and WM varies as a function of other factors. For example, the association between adversity in adulthood and WM might be stronger for people who also experienced adversity during childhood, either due to early developmental calibration to chronic stress and/or due to greater lifetime exposure to stress [28,77].

### 4.1. Strengths, limitations and future directions

This study had several strengths. First, the sample was drawn from the Dutch LISS panel, which provides a large, representative sample of the Dutch population. Second, we drew on the longitudinal nature of the LISS panel to estimate three key dimensions of adversity exposure (threat, deprivation and unpredictability), using several indicators for each. Third, we included four WM tasks, and used SEM to separate variance related to WM capacity from variance related to WM updating. This allowed us to more precisely estimate capacity and updating as two key components of WM.

This study also had limitations. First, WM updating was measured as the residual variance of a single task after accounting for WM capacity. This means that the latent WM updating measure was not a pure measure of WM updating, but also included measurement error. This decision was mainly guided by the limited number of tasks that could be included due to time constraints. To obtain a more reliable measure of WM updating, it would be better to include several different WM updating tasks, just like we used several different WM capacity tasks. Second, as this was an online study, we had only limited control over the environment in which people completed the study. The models accounted for self-reported noise and distractions, and we excluded participants who interacted with other browser tabs during the WM tasks. Yet, there may have been other, unmeasured factors that could lower the reliability of our study relative to lab-based studies. Third, our results appeared to be underpowered, despite including 759 participants, which suggests that the associations between adversity and WM in adulthood are smaller than expected based on previous literature. Finally, our study did not include genetic measures. It is well-established that genetic variation accounts for a substantial portion of the individual differences in executive functions [78]. However, for genetics to have confounded our study, it would need to have caused both individual differences in cognition and in adversity exposures—producing non-causal associations between adversity and cognition. Testing this fuller picture would require using genetically informative designs.

Future research could build on the current study in four ways. First, modelling WM ability on a latent level using multiple tasks could be applied more broadly in the field of adversity research, as studies rarely directly account for the overlap in key cognitive processes across WM tasks. This is especially important for adaptation-based research focusing on WM updating ability, as WM capacity plays a substantial role in performance on updating tasks. Second, future work is needed to better understand the role of developmental timing: is adversity experienced earlier or later in life associated differently with WM across the lifespan? Third, more research is needed to better understand the relationship between more objective (e.g. income-to-needs ratio) and subjective (e.g. perceived scarcity) indicators of adversity, as well as their respective association with cognitive functioning [79]. In our study, mean INR and mean perceived scarcity correlated moderately, suggesting that they capture similar but separable aspects of material deprivation, which could show different associations with cognition. Fourth, the field needs to account for functional heterogeneity within adversity-exposed populations [80]. In a recent study, the majority of U.S. adolescents with low socioeconomic resources performed on par with their privileged peers [81]. The deficit pattern observed in the population as a whole was driven by a much smaller, cognitively less resilient, subgroup. A valuable direction is to combine such a ‘person-centred’ approach with structural equation modelling to estimate specific WM abilities among different subgroups within adversity-exposed populations.

## 8. Conclusion

Our psychometric investigation yielded inconclusive evidence for associations between adverse experiences in adulthood and WM capacity and updating ability: differences in abilities were not significantly different from zero, yet also not negligibly small. This study is part of a recent shift in adversity research towards a more balanced view, focusing not just on cognitive deficits but also on potential adaptations. This has spurred a growing number of studies investigating more precise links between specific types of adversity and different cognitive abilities. Adaptation perspectives in particular have emphasized the need to be more precise about how specific types of adversity are associated with specific cognitive abilities. However, this increased need for precision in the measurement of cognitive abilities requires more advanced psychometric approaches. For this, adversity researchers can draw, more than they currently do, on decades of psychometric research focused on WM and other cognitive abilities. Our findings suggest that this may lead to a more complicated picture compared with traditional investigations into raw performance. However, this will ultimately lead to a better understanding of the unique abilities that develop in contexts of adversity, as well as more precise intervention targets.

## Data Availability

All scripts and materials needed to reproduce the findings are available on the article's Github repository [82]. We also include instructions on how to reproduce each step of our analyses. In this paper, we make use of data from the LISS panel (Longitudinal Internet studies for the Social Sciences) managed by the non-profit research institute Centerdata (Tilburg University, the Netherlands). All datasets are available in the LISS data archive. Researchers who want to access the data are required to sign a statement confirming that information about individual persons, households, etc., will not be released to others [83]. Supplementary material is available online [84].

## References

[1] Farah MJ, Shera DM, Savage JH, Betancourt L, Giannetta JM, Brodsky NL, Malmud EK, Hurt H. 2006 Childhood poverty: specific associations with neurocognitive development. Brain Res. **1110**, 166–174. (10.1016/j.brainres.2006.06.072) 16879809

[2] Sheridan MA *et al*. 2022 Early deprivation alters structural brain development from middle childhood to adolescence. Sci. Adv. **8**, eabn4316. (10.1126/sciadv.abn4316) 36206331 PMC9544316

[3] Sheridan MA, McLaughlin KA. 2014 Dimensions of early experience and neural development: deprivation and threat. Trends Cogn. Sci. **18**, 580–585. (10.1016/j.tics.2014.09.001) 25305194 PMC4252647

[4] Ellis BJ, Figueredo AJ, Brumbach BH, Schlomer GL. 2009 Fundamental dimensions of environmental risk : the impact of harsh versus unpredictable environments on the evolution and development of life history strategies. Hum. Nat. **20**, 204–268. (10.1007/s12110-009-9063-7) 25526958

[5] Ellis BJ, Sheridan MA, Belsky J, McLaughlin KA. 2022 Why and how does early adversity influence development? Toward an integrated model of dimensions of environmental experience. Dev. Psychopathol. **34**, 447–471. (10.1017/S0954579421001838) 35285791

[6] McLaughlin KA, Sheridan MA, Humphreys KL, Belsky J, Ellis BJ. 2021 The value of dimensional models of early experience: thinking clearly about concepts and categories. Perspect. Psychol. Sci. **16**, 1463–1472. (10.1177/1745691621992346) 34491864 PMC8563369

[7] McLaughlin KA, Sheridan MA. 2016 Beyond cumulative risk: a dimensional approach to childhood adversity. Curr. Dir. Psychol. Sci. **25**, 239–245. (10.1177/0963721416655883) 27773969 PMC5070918

[8] Oberauer K *et al*. 2018 Benchmarks for models of short-term and working memory. Psychol. Bull. **144**, 885–958. (10.1037/bul0000153) 30148379

[9] Peng P, Fuchs D. 2016 A meta-analysis of working memory deficits in children with learning difficulties: is there a difference between verbal domain and numerical domain? J. Learn. Disabil. **49**, 3–20. (10.1177/0022219414521667) 24548914

[10] Chiappe P, Siegel LS, Hasher L. 2000 Working memory, inhibitory control, and reading disability. Mem. Cognit. **28**, 8–17. (10.3758/BF03211570) 10714133

[11] Cowan N. 2014 Working memory underpins cognitive development, learning, and education. Educ. Psychol. Rev. **26**, 197–223. (10.1007/s10648-013-9246-y) 25346585 PMC4207727

[12] Conway ARA, Kane MJ, Engle RW. 2003 Working memory capacity and its relation to general intelligence. Trends Cogn. Sci. **7**, 547–552. (10.1016/j.tics.2003.10.005) 14643371

[13] Mutter B, Alcorn MB, Welsh M. 2006 Theory of mind and executive function: working-memory capacity and inhibitory control as predictors of false-belief task performance. Percept. Mot. Skills. **102**, 819–835. (10.2466/pms.102.3.819-835) 16916162

[14] Ahmed SF, Tang S, Waters NE, Davis-Kean P. Executive function and academic achievement: longitudinal relations from early childhood to adolescence. J. Educ. Psychol. **111**, 446–458. (10.1037/edu0000296)

[15] Alloway TP, Alloway RG. 2010 Investigating the predictive roles of working memory and IQ in academic attainment. J. Exp. Child Psychol. **106**, 20–29. (10.1016/j.jecp.2009.11.003) 20018296

[16] Guo Z, Zou J, He C, Tan X, Chen C, Feng G. 2020 The importance of cognitive and mental factors on prediction of job performance in Chinese high-speed railway dispatchers. J. Adv. Transp. **2020**, 1–13. (10.1155/2020/7153972)

[17] Spiegel JA, Goodrich JM, Morris BM, Osborne CM, Lonigan CJ. 2021 Relations between executive functions and academic outcomes in elementary school children: a meta-analysis. Psychol. Bull. **147**, 329–351. (10.1037/bul0000322) 34166004 PMC8238326

[18] Goodman JB, Freeman EE, Chalmers KA. 2019 The relationship between early life stress and working memory in adulthood: a systematic review and meta-analysis. Memory **27**, 868–880. (10.1080/09658211.2018.1561897) 30588865

[19] Duval ER, Garfinkel SN, Swain JE, Evans GW, Blackburn EK, Angstadt M, Sripada CS, Liberzon I. 2017 Childhood poverty is associated with altered hippocampal function and visuospatial memory in adulthood. Dev. Cogn. Neurosci. **23**, 39–44. (10.1016/j.dcn.2016.11.006) 28011437 PMC5253253

[20] Hanson JL, Chung MK, Avants BB, Rudolph KD, Shirtcliff EA, Gee JC, Davidson RJ, Pollak SD. 2012 Structural variations in prefrontal cortex mediate the relationship between early childhood stress and spatial working memory. J. Neurosci. **32**, 7917–7925. (10.1523/JNEUROSCI.0307-12.2012) 22674267 PMC3375595

[21] Bos KJ, Fox N, Zeanah CH, Nelson Iii CA. 2009 Effects of early psychosocial deprivation on the development of memory and executive function. Front. Behav. Neurosci. **3**, 16. (10.3389/neuro.08.016.2009) 19750200 PMC2741295

[22] Evans GW, Schamberg MA. 2009 Childhood poverty, chronic stress, and adult working memory. Proc. Natl Acad. Sci. USA **106**, 6545–6549. (10.1073/pnas.0811910106) 19332779 PMC2662958

[23] Hackman DA, Farah MJ, Meaney MJ. 2010 Socioeconomic status and the brain: mechanistic insights from human and animal research. Nat. Rev. Neurosci. **11**, 651–659. (10.1038/nrn2897) 20725096 PMC2950073

[24] Noble KG, McCandliss BD, Farah MJ. 2007 Socioeconomic gradients predict individual differences in neurocognitive abilities. Dev. Sci. **10**, 464–480. (10.1111/j.1467-7687.2007.00600.x) 17552936

[25] Nweze T, Nwoke MB, Nwufo JI, Aniekwu RI, Lange F. 2021 Working for the future: parentally deprived nigerian children have enhanced working memory ability. J. Child Psychol. Psychiatry. **62**, 280–288. (10.1111/jcpp.13241) 32302431

[26] Klein K, Boals A. 2001 The relationship of life event stress and working memory capacity. Appl. Cogn. Psychol. **15**, 565–579. (10.1002/acp.727)

[27] Shields GS, Ramey MM, Slavich GM, Yonelinas AP. 2019 Determining the mechanisms through which recent life stress predicts working memory impairments: precision or capacity? Stress **22**, 280–285. (10.1080/10253890.2018.1556635) 30767585 PMC6476640

[28] Shields GS, Slavich GM. 2017 Lifetime stress exposure and health: a review of contemporary assessment methods and biological mechanisms. Soc. Personal. Psychol. Compass **11**, e12335. (10.1111/spc3.12335) 28804509 PMC5552071

[29] Frost A, Moussaoui S, Kaur J, Aziz S, Fukuda K, Niemeier M. 2021 Is the n-back task a measure of unstructured working memory capacity? Towards understanding its connection to other working memory tasks. Acta Psychol. **219**, 103398. (10.1016/j.actpsy.2021.103398) 34419689

[30] Kane MJ, Conway ARA, Miura TK, Colflesh GJH. 2007 Working memory, attention control, and the n-back task: a question of construct validity. J. Exp. Psychol. Learn. Mem. Cogn. **33**, 615–622. (10.1037/0278-7393.33.3.615) 17470009

[31] Young ES, Frankenhuis WE, DelPriore DJ, Ellis BJ. 2022 Hidden talents in context: cognitive performance with abstract versus ecological stimuli among adversity-exposed youth. Child Dev. **93**, 1493–1510. (10.1111/cdev.13766) 35404500 PMC9543758

[32] Young ES, Griskevicius V, Simpson JA, Waters TEA, Mittal C. 2018 Can an unpredictable childhood environment enhance working memory? Testing the sensitized-specialization hypothesis. J. Pers. Soc. Psychol. **114**, 891–908. (10.1037/pspi0000124) 29389153

[33] Ellis BJ, Bianchi J, Griskevicius V, Frankenhuis WE. 2017 Beyond risk and protective factors: an adaptation-based approach to resilience. Perspect. Psychol. Sci. **12**, 561–587. (10.1177/1745691617693054) 28679332

[34] Frankenhuis WE, Young ES, Ellis BJ. 2020 The hidden talents approach: theoretical and methodological challenges. Trends Cogn. Sci. **24**, 569–581. (10.1016/j.tics.2020.03.007) 32360117

[35] Frankenhuis WE, de Weerth C. 2013 Does early-life exposure to stress shape or impair cognition? Curr. Dir. Psychol. Sci. **22**, 407–412. (10.1177/0963721413484324)

[36] Fields A *et al*. 2021 Adaptation in the face of adversity: decrements and enhancements in children’s cognitive control behavior following early caregiving instability. Dev. Sci. **24**, e13133. (10.1111/desc.13133) 34080760 PMC8530827

[37] Mittal C, Griskevicius V, Simpson JA, Sung S, Young ES. 2015 Cognitive adaptations to stressful environments: when childhood adversity enhances adult executive function. J. Pers. Soc. Psychol. **109**, 604–621. (10.1037/pspi0000028) 26414842

[38] Steudte-Schmiedgen S, Stalder T, Kirschbaum C, Weber F, Hoyer J, Plessow F. 2014 Trauma exposure is associated with increased context-dependent adjustments of cognitive control in patients with posttraumatic stress disorder and healthy controls. Cogn. Affect. Behav. Neurosci. **14**, 1310–1319. (10.3758/s13415-014-0299-2) 24888985

[39] Frischkorn GT, von Bastian CC, Souza AS, Oberauer K. 2022 Individual differences in updating are not related to reasoning ability and working memory capacity. J. Exp. Psychol. Gen. **151**, 1341–1357. (10.1037/xge0001141) 35201837

[40] Löffler C, Frischkorn GT, Hagemann D, Sadus K, Schubert AL. 2024 The common factor of executive functions measures nothing but speed of information uptake. Psychol. Res. **88**, 1092–1114. (10.1007/s00426-023-01924-7) 38372769 PMC11143038

[41] Gruszka A, Nęcka E. 2017 Limitations of working memory capacity: the cognitive and social consequences. Eur. Manag. J. **35**, 776–784. (10.1016/j.emj.2017.07.001)

[42] Oberauer K. 2009 Design for a working memory. In Psychology of learning and motivation (edRoss BHpp. 45–100, vol. 51. Elsevier. (10.1016/S0079-7421(09)51002-X)

[43] Wilhelm O, Hildebrandt A, Oberauer K. 2013 What is working memory capacity, and how can we measure it? Front. Psychol. **4**, 433. (10.3389/fpsyg.2013.00433) 23898309 PMC3721021

[44] Oberauer K. 2019 Working memory capacity limits memory for bindings. J. Cogn. **2**, 40. (10.5334/joc.86) 31576379 PMC6753309

[45] Oberauer K, Süß HM, Schulze R, Wilhelm O, Wittmann WW. 2000 Working memory capacity — facets of a cognitive ability construct. Pers. Individ. Dif. **29**, 1017–1045. (10.1016/S0191-8869(99)00251-2)

[46] Oberauer K. 2005 Binding and inhibition in working memory: individual and age differences in short-term recognition. J. Exp. Psychol. Gen. **134**, 368–387. (10.1037/0096-3445.134.3.368) 16131269

[47] Ecker UKH, Lewandowsky S, Oberauer K, Chee AEH. 2010 The components of working memory updating: an experimental decomposition and individual differences. J. Exp. Psychol. Learn. Mem. Cogn. **36**, 170–189. (10.1037/a0017891) 20053053

[48] Schmiedek F, Hildebrandt A, Lövdén M, Wilhelm O, Lindenberger U. 2009 Complex span versus updating tasks of working memory: the gap is not that deep. J. Exp. Psychol. Learn. Mem. Cogn. **35**, 1089–1096. (10.1037/a0015730) 19586272

[49] Walasek N, Young ES, Frankenhuis WE. 2024 A framework for studying environmental statistics in developmental science. Psychol. Methods (10.1037/met0000651) 39023977

[50] Lakens D, Scheel AM, Isager PM. 2018 Equivalence testing for psychological research: a tutorial. Adv. Methods Pract. Psychol. Sci. **1**, 259–269. (10.1177/2515245918770963)

[51] Sheridan MA, Shi F, Miller AB, Salhi C, McLaughlin KA. 2020 Network structure reveals clusters of associations between childhood adversities and development outcomes. Dev. Sci. **23**, e12934. (10.1111/desc.12934) 31869484 PMC7308216

[52] Salhi C, Beatriz E, McBain R, McCoy D, Sheridan M, Fink G. 2021 Physical discipline, deprivation, and differential risk of developmental delay across 17 countries. J. Am. Acad. Child Adolesc. Psychiatry **60**, 296–306. (10.1016/j.jaac.2020.02.016) 32201317

[53] Bradley RH, Corwyn RF, McAdoo HP, García Coll C. 2001 The home environments of children in the United States part I: variations by age, ethnicity, and poverty status. Child Dev. **72**, 1844–1867. (10.1111/1467-8624.t01-1-00382) 11768149

[54] Lurie LA, Rosen ML, Weissman DG, Machlin L, Lengua L, Sheridan MA, McLaughlin KA. 2024 Cognitive stimulation as a mechanism linking socioeconomic status and neural function supporting working memory: a longitudinal fMRI study. Cereb. Cortex **34**, bhad545. (10.1093/cercor/bhad545) 38236725 PMC11486689

[55] Rosen ML, Amso D, McLaughlin KA. 2019 The role of the visual association cortex in scaffolding prefrontal cortex development: a novel mechanism linking socioeconomic status and executive function. Dev. Cogn. Neurosci. **39**, 100699. (10.1016/j.dcn.2019.100699) 31446376 PMC6783336

[56] Scherpenzeel A. 2011 Data collection in a probability-based internet panel: how the liss panel was built and how it can be used. Bull. Sociol. Methodol. **109**, 56–61. (10.1177/0759106310387713)

[57] Kretzschmar A, Gignac GE. 2019 At what sample size do latent variable correlations stabilize? J. Res. Pers. **80**, 17–22. (10.1016/j.jrp.2019.03.007)

[58] Frankenhuis WE, Bijlstra G. 2018 Does exposure to hostile environments predict enhanced emotion detection? Collabra Psychol. **4**, 18. (10.1525/collabra.127)

[59] Sweeten G. 2012 Scaling criminal offending. J. Quant. Criminol. **28**, 533–557. (10.1007/s10940-011-9160-8)

[60] Brakel M *et al*. 2023 Op weg naar een nieuwe armoedegrens. Tussenrapport van het gezamenlijke project ’Uniformering armoedeafbakening’. See https://www.scp.nl/publicaties/publicaties/2023/06/30/op-weg-naar-een-nieuwe-armoedegrens.

[61] Key N, Prager D, Burns C. 2017 Farm household income volatility: An analysis using panel data from a national survey. US Department of Agriculture, Economic Research Service. See https://www.ers.usda.gov/publications/pub-details/?pubid=82563.

[62] Liu S, Zalewski M, Lengua L, Gunnar MR, Giuliani N, Fisher PA. 2022 Material hardship level and unpredictability in relation to US households’ family interactions and emotional well-being: insights from the COVID-19 pandemic. Soc. Sci. Med. **307**, 115173. (10.1016/j.socscimed.2022.115173) 35785642 PMC9242702

[63] Ugarte E, Hastings PD. 2023 Assessing unpredictability in caregiver-child relationships: insights from theoretical and empirical perspectives. Dev. Psychopathol. 1–20. (10.1017/S0954579423000305) 37017124

[64] Young ES, Frankenhuis WE, Ellis BJ. 2020 Theory and measurement of environmental unpredictability. Evol. Hum. Behav. **41**, 550–556. (10.1016/j.evolhumbehav.2020.08.006)

[65] Li Z, Liu S, Hartman S, Belsky J. 2018 Interactive effects of early-life income harshness and unpredictability on children’s socioemotional and academic functioning in kindergarten and adolescence. Dev. Psychol. **54**, 2101–2112. (10.1037/dev0000601) 30265037

[66] Young ES, Vermeent S, Frankenhuis WE, Nivison M, Simpson JA, Roisman GI. 2024 How does adversity shape performance across different abilities in the same person? Dev. Psychopathol. 1–18. (10.1017/S0954579424001433) 39310942

[67] Conway ARA, Kane MJ, Bunting MF, Hambrick DZ, Wilhelm O, Engle RW. 2005 Working memory span tasks: a methodological review and user’s guide. Psychon. Bull. Rev. **12**, 769–786. (10.3758/BF03196772) 16523997

[68] Vermeent S. 2023 Projectlog: tools for documenting your project workflow. See https://stefanvermeent.github.io/projectlog/.

[69] Rosseel Y. 2012 Lavaan: an R package for structural equation modeling. J. Stat. Softw. **48**, 1–36. (10.18637/jss.v048.i02)

[70] Oberski D. 2014 Lavaan.survey: an R package for complex survey analysis of structural equation models. J. Stat. Softw. **57**. (10.18637/jss.v057.i01)

[71] Hu L, Bentler PM. 1999 Cutoff criteria for fit indexes in covariance structure analysis: conventional criteria versus new alternatives. Struct. Equ. Modeling **6**, 1–55. (10.1080/10705519909540118)

[72] Benjamini Y, Hochberg Y. 1995 Controlling the false discovery rate: a practical and powerful approach to multiple testing. J. R. Stat. Soc. Ser. B **57**, 289–300. (10.1111/j.2517-6161.1995.tb02031.x)

[73] Cribbie RA. 2007 Multiplicity control in structural equation modeling. Struct. Equ. Modeling **14**, 98–112. (10.1080/10705510709336738)

[74] Burnham KP, Anderson DR (eds). 2002 Model selection and multimodel inference: a practical information-theoretic approach. New York: Springer. See http://link.springer.com/10.1007/978-0-387-22456-5_6.

[75] Hoijtink H, Mulder J, van Lissa C, Gu X. 2019 A tutorial on testing hypotheses using the bayes factor. Psychol. Methods **24**, 539–556. (10.1037/met0000201) 30742472

[76] Lewandowsky S, Oberauer K, Yang LX, Ecker UKH. 2010 A working memory test battery for MATLAB. Behav. Res. Methods **42**, 571–585. (10.3758/BRM.42.2.571) 20479189

[77] Hostinar CE, Gunnar MR. 2013 The developmental effects of early life stress: an overview of current theoretical frameworks. Curr. Dir. Psychol. Sci. **22**, 400–406. (10.1177/0963721413488889) 25419054 PMC4236853

[78] Friedman NP, Miyake A, Young SE, DeFries JC, Corley RP, Hewitt JK. 2008 Individual differences in executive functions are almost entirely genetic in origin. J. Exp. Psychol. **137**, 201–225. (10.1037/0096-3445.137.2.201) PMC2762790 18473654

[79] Smith KE, Pollak SD. 2021 Rethinking concepts and categories for understanding the neurodevelopmental effects of childhood adversity. Perspect. Psychol. Sci. **16**, 67–93. (10.1177/1745691620920725) 32668190 PMC7809338

[80] Masten AS. 2001 Ordinary magic: resilience processes in development. Am. Psychol. **56**, 227–238. (10.1037//0003-066x.56.3.227) 11315249

[81] Shariq D, Romeo RR, Gard AM. 2024 Cognitive resilience and vulnerability to socioeconomic disadvantage: individual, family, school, and neighborhood predictors. OSF. See 10.31234/osf.io/2kew7. PMC12696620 41376539

[82] Vermeent S. 2024 GitHub. See https://stefanvermeent.github.io/liss_wm_profiles_2023.

[83] Data Statements. See https://statements.centerdata.nl/.

[84] Vermeent S, Schubert AL, DeJoseph ML, Denissen JJA, van Gelder JL, Frankenhuis WE. 2024. Supplementary material from: Inconclusive evidence for associations between adverse experiences in adulthood and working memory performance. FigShare (10.6084/m9.figshare.c.7569523) PMC11706643 39780975

