# Correction to: ‘Inconclusive evidence for associations between adverse experiences in adulthood and working memory performance’ (2025), by Vermeent *et al.*

**DOI:** 10.1098/rsos.250722

**Published:** 2025-06-11

**Authors:** Stefan Vermeent, Anna-Lena Schubert, Meriah L. DeJoseph, Jaap J. A. Denissen, Jean-Louis van Gelder, Willem E. Frankenhuis

**Affiliations:** ^1^ Evolutionary and Population Biology, Institute for Biodiversity and Ecosystem Dynamics, University of Amsterdam, Amsterdam, The Netherlands; ^2^ Department of Psychology, University of Mainz, Mainz, Germany; ^3^ Stanford University, Stanford, CA, USA; ^4^ Department of Psychology, Utrecht University, Utrecht, The Netherlands; ^5^ Max Planck Institute for the Study of Crime, Security and Law, Freiburg, Germany

**Keywords:** working memory, adversity, deficits, developmental adaptation, structural equation modelling

In reference to Vermeent, S., Schubert, A.-L., DeJoseph, M.L., Denissen, J.J.A., van Gelder, J.-L., & Frankenhuis, W.E. (2025). Inconclusive evidence for associations between adverse experiences in adulthood and working memory performance. *Royal Society Open Science, 12*(1). https://doi.org/10.1098/rsos.241837.

In March 2025, a colleague discovered a programming bug in the code used to analyse the data. This bug affected the computation of the perceived scarcity composite. The variable name of one of the items in this composite from the LISS archive (*Can you indicate, on a scale from 0 to 10, how hard or how easy it is for you to live off your income*) changed in wave 12. This name change was not handled in our code, and as a consequence, our measure only included responses between 2008 and 2018 (waves 1–11) but not responses between 2019 and 2023 (waves 12–16). After discovering the programming bug, we updated our scripts and repeated all the analyses. This led to a few minor changes to our results, although our general conclusions remain the same. We first provide a general summary of the changes, and then we quote the changes in the manuscript separately by section.

## General summary of changes

(1) The bug fix led to only one change in confirmatory analyses. The association between perceived scarcity and WM capacity changed from ‘inconclusive’ (neither different from zero nor statistically equivalent to zero) to being practically equivalent to zero. This change did not influence our substantive conclusions. The updated results still do not provide evidence for either deficit or adaptation frameworks. We have edited the Abstract, Results, Discussion and figure 5 to reflect this change.
(2) The bug fix also led to changes in exploratory analyses. First, threat is no longer associated with performance on the Binding, Operation Span and Rotation Span tasks, and two associations changed from being inconclusive to being practically equivalent to zero. Second, the Bayes factors for testing practical equivalence in the electronic supplementary materials changed slightly. We now find moderate (rather than strong) evidence in favour of the hypothesis that the association between mean income-to-needs ratio and WM updating falls within the equivalence bounds. All other interpretations of Bayes factors remain the same. We have edited the Results and electronic supplementary materials in line with these changes.
(3) The bug fix also led to small changes in some correlations between independent variables, SEM fit statistics and regression coefficients of other independent variables in the models involving perceived scarcity. None of these changes led to changes in direction of association, statistical significance or model selection. We have edited the Methods, Results and table 2 in line with these changes.

**Figure 5.**
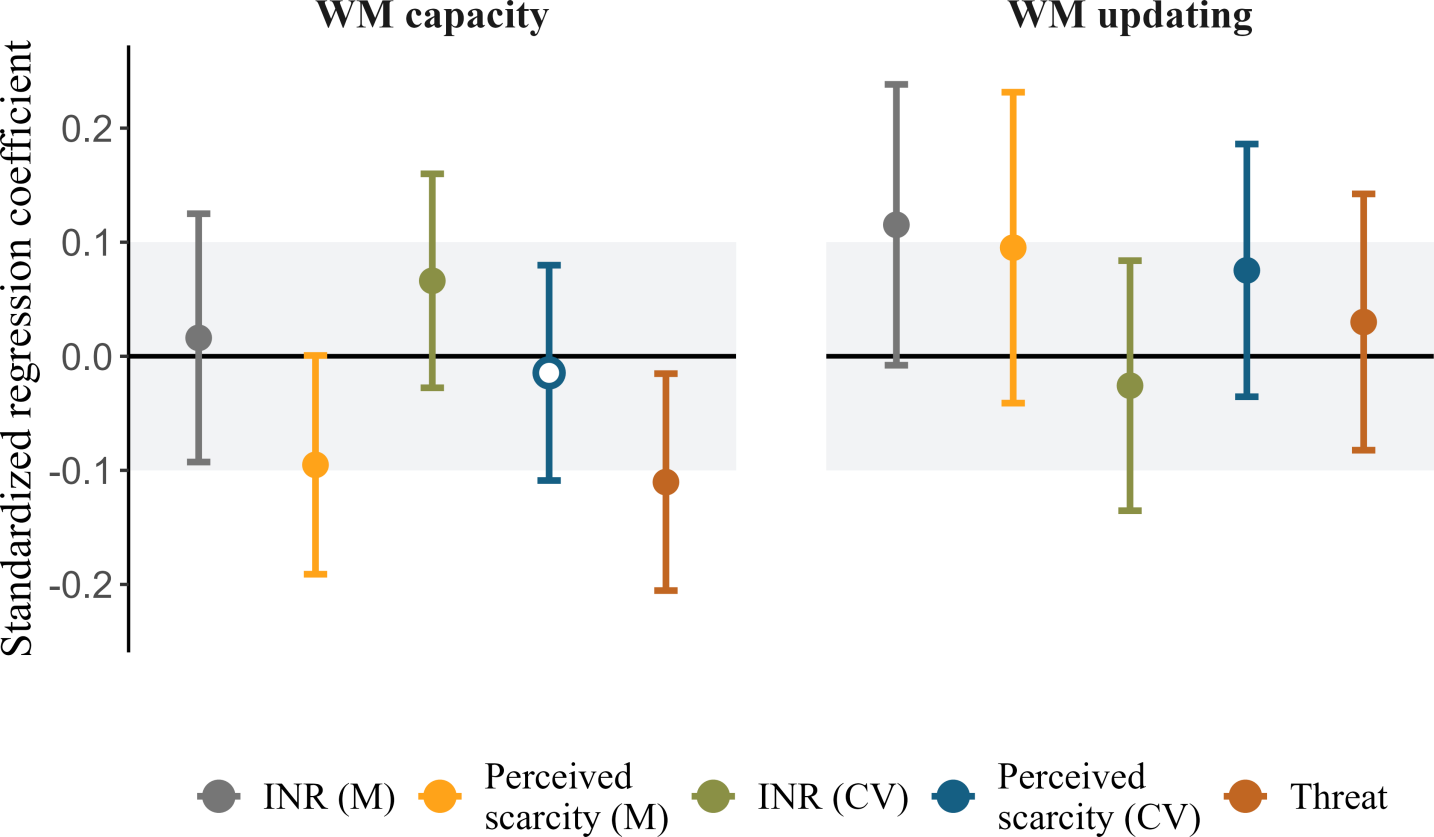
Results of the structural part of the SEM model testing the association between threat, deprivation and unpredictability on latent estimates of WM capacity and WM updating. The grey area shows the area of practical equivalence. Solid points indicate effects outside the area of practical equivalence, which was true for all effects. Standard errors represent the 95% confidence intervals. CV = coefficient of variation; INR = income-to-needs ratio; M = mean; WM = working memory.

**Table 2.** Spearman correlation between the main independent variables.

	1	2	3	4	5	6	7	8	9	10	11	12	13	14
INR (M)	—													
living off income (M)	−0.51***	—												
financial troubles (M)	−0.43***	0.68***	—											
current situation (M)	−0.51***	0.79***	0.69***	—										
perceived scarcity (M)	−0.54***	0.97***	0.77***	0.91***	—									
INR (CV)	−0.17***	0.09*	0.21***	0.14***	0.13***	—								
living off income (CV)	0.21***	−0.35***	−0.01	−0.27***	−0.31***	0.20***	—							
financial troubles (CV)	−0.36***	0.60***	0.92***	0.60***	0.68***	0.24***	0.07	—						
current situation (CV)	0.20***	−0.18***	0.04	−0.11**	−0.14***	0.12**	0.39***	0.13***	—					
perceived scarcity (CV)	0.18***	−0.17***	0.12**	−0.09*	−0.11**	0.16***	0.55***	0.22***	0.97***	—				
neighbourhood safety	−0.13***	0.20***	0.16***	0.17***	0.20***	0.05	−0.06	0.12**	−0.05	−0.05	—			
neighbourhood violence scale	−0.22***	0.28***	0.19***	0.22***	0.27***	0.02	−0.17***	0.16***	−0.06	−0.07	0.24***	—		
crime victimization	0.01	0.10**	0.18***	0.16***	0.13***	0.10**	0.03	0.17***	0.07	0.08*	0.06	0.12**	—	
threat	−0.21***	0.31***	0.24***	0.26***	0.31***	0.07	−0.15***	0.20***	−0.06	−0.06	0.58***	0.89***	0.26***	—
mean	1.99	4.04	1.30	2.35	2.56	0.22	0.30	0.21	0.27	−0.01	1.45	2.39	1.04	−0.02
s.d.	0.76	1.48	0.53	0.75	0.84	0.19	0.16	0.24	0.15	0.84	1.47	0.95	1.27	0.68
min	0.09	1.00	1.00	1.00	1.00	0.01	0.00	0.00	0.00	−1.70	0.00	1.00	0.00	−1.07
max	6.10	9.56	4.44	5.00	6.11	1.52	0.94	0.92	0.93	3.43	8.00	6.86	7.00	3.68
skew	1.06	0.52	2.47	0.44	0.78	2.31	0.86	0.62	0.22	0.33	1.18	1.33	1.28	1.21
kurtosis	3.42	0.48	6.86	−0.08	0.75	8.83	0.85	−1.01	0.87	0.95	1.22	2.35	1.27	2.05

*Note:* *p < 0.05, **p < 0.01, ***p < 0.001.

CV, coefficient of variance; INR, income-to-needs ratio; M, mean; Perc. Scarcity, perceived scarcity.

## Overview of changes per section

### Changes to ‘Abstract’

We did not find associations between adversity and WM capacity or updating, and only found practical equivalence for unpredictability in perceived scarcity with WM capacity.

### Changes to ‘Methods’

#### 2.2.3 Unpredictability

The perceived unpredictability component was almost fully determined by the item about people’s current situation (0.82), followed by difficulties to live off income (0.45) and financial troubles (0.22).

### Changes to ‘Results’

#### 6.1.2 Associations between adversity and WM

None of the adversity measures were significantly associated with WM capacity after adjusting for multiple testing (all *p*s ≥ 0.115). We also did not find evidence for practical equivalence for associations between any of the adversity measures and WM capacity (all *p*s ≥ 0.038). Similarly, none of the adversity measures were significantly associated with WM updating after adjusting for multiple testing (all *p*s ≥ 0.305). We also did not find evidence for practical equivalence to zero for associations between any of the adversity measures and WM updating (all *p*s ≥ 0.092).

### 6.2 Post hoc non-preregistered analyses

Threat had small, significant negative associations with performance on the Rotation Span Task (β = −0.10, *p* = 0.013), Operation Span Task (β = −0.11, *p* = 0.004) and Binding Task (β = −0.09, *p* = 0.025). None of the types of adversity were significantly associated with performance on the Updating Task (all *p*s > 0.299), and only the association with unpredictability in the income-to-needs was practically equivalent to zero (*p* = 0.035). Constraining all paths to latent WM capacity to zero significantly reduced model fit, although the change in AIC was below the cut-off as proposed by [74], ΔAIC = 9.43, Δχ(5) = 16.10, *p* = 0.007. Constraining all paths to latent WM updating did not significantly reduce model fit, ΔAIC = 2.68, Δχ(5) = 6.77, *p* = 0.238, Robust CFI = 1, robust RMSEA = 0.02, 95% CI = [0, 0.03]. For all but one association, the model comparisons showed at least strong evidence in favour of the data being more likely under the hypothesis that the effects fell within the equivalence bounds (BF_10_ ranging between 9.3 and 225). The only exception was the association between threat and WM capacity, for which we found moderate evidence in favour of the data being more likely under the hypothesis that the effect fell within the equivalence bounds (BF_10_ = 7.7).

## Changes to ‘Discussion’

On the other hand, only the association between unpredictability in perceived scarcity and WM capacity fell within the pre-specified region of practical equivalence to zero (i.e., a between-person difference in performance that is practically equivalent to zero).

These findings are largely inconclusive, as we also did not find evidence for practical equivalence in our preregistered analysis, except for the association between unpredictability in perceived scarcity and WM capacity.

## Changes to the electronic supplementary materials

(https://doi.org/10.6084/m9.figshare.27958372.v2).

